# Quantification of diversity sampling bias resulting from rice root bacterial isolation on popular and nitrogen-free culture media using 16S amplicon barcoding

**DOI:** 10.1371/journal.pone.0279049

**Published:** 2023-04-06

**Authors:** Moussa Sondo, Issa Wonni, Agnieszka Klonowska, Kadidia Koïta, Lionel Moulin

**Affiliations:** 1 PHIM Plant Health Institute, IRD, CIRAD, INRAE, Institut Agro, Univ Montpellier, Montpellier, France; 2 Ecole doctorale sciences et Technologie, Biologie végétale, Phytopathologie, Université Joseph Ki Zerbo, Ouagadougou, Burkina Faso; 3 Institut de l’Environnement et de Recherches Agricoles (INERA), Bobo-Dioulasso, Burkina Faso; 4 LMI Pathobios, Observatoire des Agents Phytopathogènes en Afrique de l’Ouest, Bobo-Dioulasso, Burkina Faso; National Taiwan University, TAIWAN

## Abstract

Culturing bacteria from plant material is well known to be conducive to strong bias compared to the actual diversity in the original samples. This bias is related to the bacterial cultivability, chemical composition of the media and culture conditions. Recovery bias is often observed but has never been quantified on different media using an amplicon barcoding approach whereby plant microbiota DNA extractions are compared to DNA extracted from serial dilutions of the same plant tissues grown on bacterial culture media. In this study, we: i) quantified the bacterial culturing diversity bias using 16S amplicon barcode sequencing by comparing a culture-dependent approach (CDA) focused on rice roots on four commonly used bacterial media (10% and 50% TSA, plant-based medium with rice flour, nitrogen free medium NGN and NFb) versus a culture-independent approach (CIA) assessed with DNA extracted directly from root and rhizosphere samples; ii) assessed enriched and missing taxa detected on the different media; iii) used biostatistics functional predictions to highlight metabolic profiles that could potentially be enriched in the CDA and CIA. A comparative analysis of the two approaches revealed that among the 22 phyla present in microbiota of the studied rice root samples, only five were present in the CDA (Proteobacteria, Firmicutes, Bacteroidetes, Actinobacteria, Verrucomicrobia). The Proteobacteria phylum was the most abundant in all CDA samples, showing high gamma-Proteobacteria enrichment. The diversity of the combined culture media represented about a third of the total microbiota diversity, and its genus diversity and frequency was documented. The functional prediction tool (PICRUSt2) detected nitrogenase enzyme enrichment in bacterial taxa sampled from nitrogen-free media, thus validating its predictive capacity. Further functional predictions also showed that the CDA mostly missed anaerobic, methylotrophic, methanotrophic and photosynthetic bacteria compared to the CIA, thereby generating valuable insight that could enable the design of *ad-hoc* culture media and conditions to increase the rice-associated microbiota cultivability.

## Introduction

Plants interact continuously with a microbiota that plays an important role in their health, fitness and productivity. In the last 10 years, the low-cost accessibility of next generation sequencing (amplicon-based sequencing and metagenomics) to scientists has enabled extensive description of the diversity of this microbiota on many model and non-model plants (e.g. in *Arabidopsis* [1] and wheat [2]). For rice, its microbiome has been widely described in different countries and rice culture practices [3–6]. This wealth of data has now provided a good overview of the main bacterial and fungal taxa inhabiting underground plant tissues (roots and rhizosphere), as well as those in their above-ground parts (phyllosphere and endosphere). The diversity determined using amplicon-barcode approaches is mainly based on fragments of ribosomal taxonomic markers such as 16S and 18S rRNA genes, with taxonomic resolution often restricted to the genus level. To access and obtain more microbial diversity and structural representativeness, several studies have been carried out using a combination of markers at different resolution levels, ranging from general (16S V3-V4 or V4 for prokaryotes, 18S V4 for microeukaryotes) to more resolutive markers (*gyrB* or *rpoB* fragments for bacteria, ITS1/ITS2 for fungi) [7–9]. Bioinformatic analysis of amplicon barcode data has also involved several novel strategies, ranging from operational taxonomic unit (OTU) clustering at different identity percentages to more advanced clustering methods using swarming algorithms [10, 11], in addition to methods inferring true amplicon sequence variants (ASV) [12].

Harnessing plant microbiota diversity with regard to plant nutrition or tolerance to pathogens, for instance, relies on the isolation and culturing of the taxonomic and/or functional diversity of the microbiota [13]. The capacity to culture and store such diversity allows us to design synthetic communities and test their various compositions on plant growth and health [14, 15]. Otherwise, different culturomics approaches have been developed to capture the bacterial diversity of plant microbiota, including culture media supplementation with various compounds, simulated natural environments, diffusion chambers, soil substrate membrane systems, isolation chips, single cell microfluidics [16], or using limiting dilutions on plates combined with dual barcode processing [17]. Substantial improvements in diversity sampling have also been achieved by popular media supplementation with plant compounds or plant-based media, while microbiologists continue to develop alternative culture methods to highlight rare and unculturable plant-associated microorganisms [16].

Several functional prediction tools, such as PICRUSt2 [18] have recently been developed to predict functional enrichment in metagenomes and even 16S amplicon barcoding data. In theory, such tools could allow the identification of metabolic functions and ecological functions that are enriched in culture-independent compared to culture-dependent approaches in order to guide culture media design or highlight culturing conditions that could help capture them [19].

It is well recognized in the microbiology community that commonly used non-selective bacterial medium, such as Lubria broth (LB), R2A, nutrient agar (NA), tryptic soy agar (TSA), are conducive to strong bias in the sampled diversity recovered from plant tissues [20, 21]. This bias has never been quantified or documented in terms of proportions using next generation sequencing (NGS) amplicon-based technologies, to the best of our knowledge. Other media, such as Norris-glucose nitrogen-free medium (NGN), nitrogen-free medium (NFb) [22], have been successfully designed to isolate dinitrogen-fixing bacteria, but proportions of recovery of the diversity of the dinitrogen-fixing community remain unclear.

In this study, we employed both culture-independent (CIA) and culture-dependent approaches (CDA) to analyse bacterial diversity in rice roots and rhizosphere soils. Specifically, we used 16S amplicon barcode sequencing to analyse DNA directly extracted from the plant samples (CIA), as well as from mass bacterial cultures of varying dilutions plated on different media (CDA), including a popular medium for isolating plant-associated bacteria (TSA at 10 and 50%), a plant-based medium (rice flour), and two nitrogen-free media (NFb, NGN). The object of this study was: i) to quantify the bias of bacterial diversity introduced by CDA compared to CIA; ii) to determine the proportions of enriched bacterial genera per medium; iii) to use functional prediction tools on amplicon data to identify specific metabolic functions or bacterial capacities present in the rice root microbiota that are missing from the CDA. Our hypothesis is that the culture-dependent approach (CDA) we used in this study, which involved high-throughput sequencing of DNA pooling from the culture media, will help overcome the issue of losing slow-growing bacteria and provide a more accurate assessment of the culturable bacterial diversity. The approach may increase the percentage recovery of bacteria and obtain a more comprehensive picture of the bacterial diversity that reflects the real diversity present in the plant samples.

## Materials and methods

### Rice root sampling and processing

**Fig 1:**
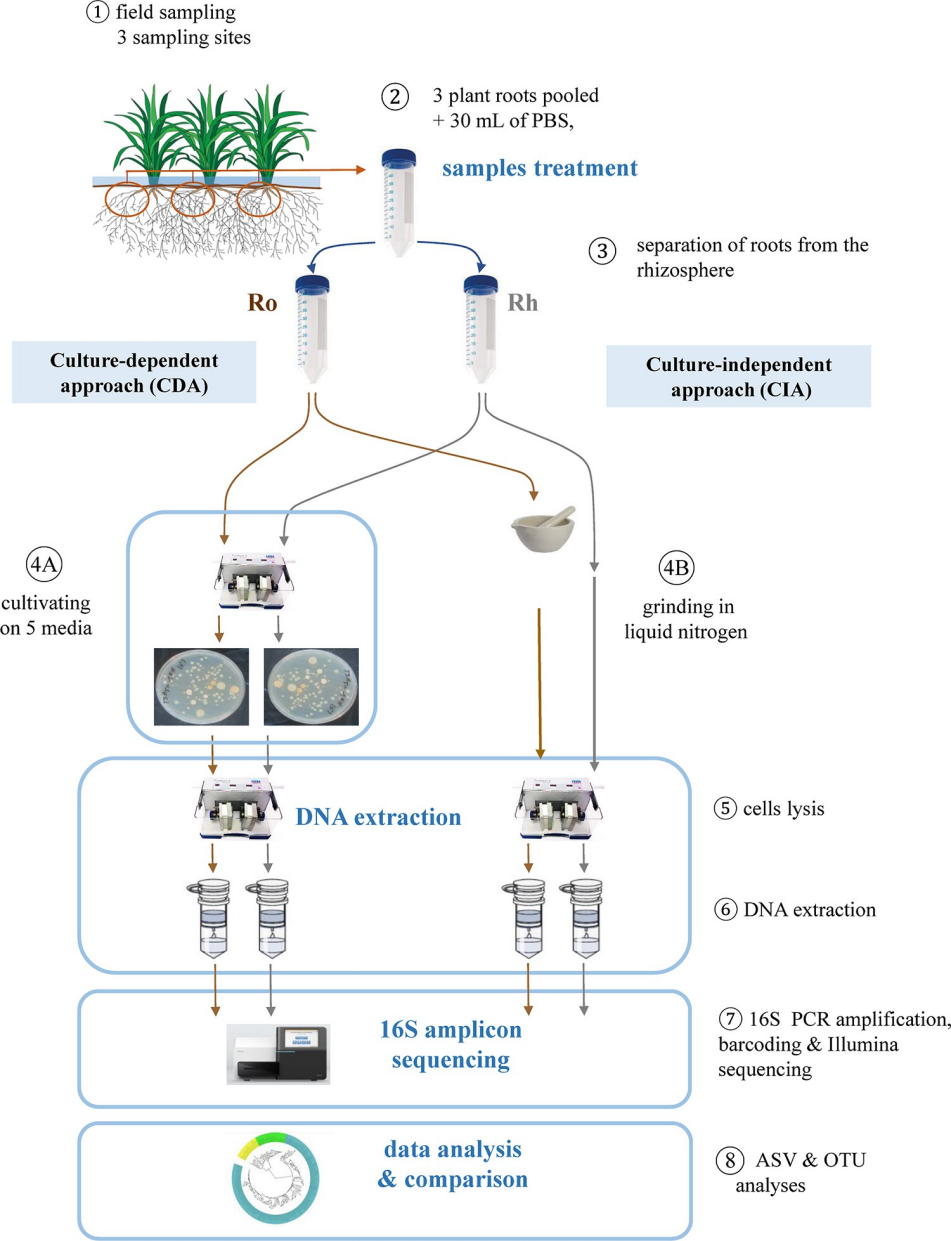
Schematic representation of the sampling and processing protocol for 16S amplicons libraries.

*Oryza sativa* ssp *indica* cv FKR64 plant roots were collected in a rice field near Bama village (western Burkina Faso, Kou Valley, 10.64384 N, -4.8302 E). This field was already assessed in a previous study and described by Barro et al. [6]. Rice sampling was authorized by a national agreement between the Burkina Faso government and farmers within the framework of a rice productivity improvement program involving INERA. Rice plants were sampled at the panicle initiation growth stage, with three sampling points chosen 10 m apart, where roots were collected from three plants (20 cm apart). Roots were hand-shaken to remove non-adherent soil. Ten roots per plant from the same sampling point were pooled to obtain three final samples in 50 mL Falcon tube containing 30 mL of sterile PBS buffer, and vortexed for 5 min to separate the rhizospheric soil from the roots. Roots were removed with sterile forceps and placed in new 50 mL Falcon tubes. From this treatment step, the rhizosphere (Rh) and roots (Ro) samples were manipulated separately (Fig 1). The rhizosphere soil in PBS was vortexed for 10 sec and then two samples of 1 mL of the rhizosphere suspension were taken after 15 sec and placed in two separate 2 mL Eppendorf tubes to be used in bacterial culture-dependent (CDA) or culture-independent approaches (CIA) for diversity estimation by a direct 16S amplicon barcoding approach. Similarly, washed roots were cut into 2 cm fragments, and then divided and placed in two 2 mL Eppendorf tubes for CDA and CIA assessment.

### Bacterial culture isolation media

Four culture media with different carbon and nitrogen sources were used to maximize the isolated bacterial diversity. First, non-selective tryptic soy agar (TSA, Sigma) medium was used at 10% (TSA10) and 50% (TSA50) concentration. It contained digests of casein and soybean meal, NaCl and agar. In addition, two nitrogen-free media were used for the isolation of potential nitrogen fixers, semi-solid NFb [22] and Norris glucose nitrogen-free medium (NGN, M712, [23]). NFb was used as semi-solid medium, which allows the development and growth of free nitrogen-fixing bacteria, due to their growth at an optimal distance for micro-aerobic conditions favourable for nitrogen fixation [22]. Finally, we included a plant-based medium, rice flour (RF), which is commonly used for isolation of fungal rice pathogens [24]. The compositions of the above culture media were as follows: TSA 10% (g/L): 0.5 NaCl, 1.7 pancreatic digest of casein, 0.3 papaic digest of soybean meal, 0.25 dextrose, 0.25 K_2_HPO_4_; NGN (g/L), 1.0 K_2_HPO_4_, 1.0 CaCO_3_, 0.2 NaCl, 0.20 MgSO_4_·7H_2_O, 0.01 FeSO_4_·7H_2_O O, 0.005 Na_2_MoO_4_·2H2O, with a glucose carbon source (10 g/L) at pH 7; NFb: (g/L), 0.5 K_2_HPO_4_, 0.2 MgSO_4_.7H2O, 0.1 NaCl, 0.02 CaCl_2_. 2H2O, 4.5 KOH, 5 malic acid, 2 mL of micronutrient solution ((g/L) 0.04 CuSO_4_.5H2O, 0.12 ZnSO_4_.7H2O, 1.40 H_3_BO_3_, 1.0 Na2MoO4.2H2O, 1.175 MnSO_4_. H2O), 2 mL of bromothymol blue (5 g/L in 0.2 N KOH), 4 mL of Fe-EDTA (solution 16.4 g/L), 1 mL of vitamin solution ((mg/0.1 L) 10 biotin, 20 pyridoxal-HCl) and pH adjusted to 6.5; RF (g/L): 20 rice flour (prepared from seeds from the FKR64 rice variety), 2.5 yeast extract. Solid and the semi-solid media were obtained by adding 2% and 0.16% g of agar, respectively.

### Culture-dependent (CDA) and independent (CIA) approaches

For the CDA, roots (200 mg) and rhizosphere soil (200 mg) were transferred into PowerBead Tubes from the DNeasy PowerSoil kit (QIAGEN) where 1 mL of PBS buffer was added, and homogenized in a TissueLyser II (QIAGEN) for 2 min (Fig 1). Dilutions (10^−2^ to 10^−5^) were performed and 50 μL of each dilution were spread on solid culture media (TSA 10%, TSA 50%, NGN, RF). For NFb medium, 50 μL of the 10^−1^ root and rhizosphere soil suspensions were inoculated in 20 mL tubes containing 10 mL of NFb semi-solid medium. Each dilution was inoculated (on plates or in tubes) with 4 replicates. After 2 to 5 days of incubation (depending on the culture medium) at 28°C, plates were examined and dilutions selected for further processing (details in S1 Table). For selected dilutions, cultivable bacteria were recovered from petri plates by adding 1 mL of sterile distilled water, scraping and mixing bacterial colonies. Bacterial suspensions obtained from the same dilution plates were collected with a pipette and transferred to sterile 15 mL Falcon tubes. For the NFb medium, bacteria which had grown in a ring shape 0.2–0.3 cm below the surface of the medium were collected. Bacterial suspensions were stored at -20°C until DNA extraction. The number of cultivable bacteria in the obtained suspensions was roughly estimated by measuring the optical density (OD) at 600 nm for all suspensions and adjusted to 10^6^ (assuming that OD600 nm of 1 corresponds to 1x10^8^ bacteria/mL). The volumes collected from the samples were centrifuged 10 min at 14,000 rpm, and the pellets obtained were used for DNA extraction.

For the culture-independent approach (CIA), pooled roots were homogenized in liquid nitrogen using a mortar and pestle, while the pooled rhizosphere samples were used directly for DNA extraction (Fig 1). A mass of 250 mg was used for DNA extraction from both sample types.

### DNA extraction

Cultivable bacteria suspensions (≈ 10^6^ cells) and ground roots and rhizospheres soil (250 mg) were transferred to PowerBead tubes (DNeasy PowerSoil, Qiagen) containing C1 buffer and homogenised in a TissueLyser II (Qiagen) at 240 rpm for 2 x 1 min. Extraction was then performed according to the protocol provided by the supplier.

### 16S amplicon-barcoding data production

Quality control of DNA, PCR amplification, library construction and MiSeq Illumina sequencing were performed by Macrogen (Seoul, South Korea) using 337F (16S_337F, 5’-GACTCCTACGGGAGGCWGCAG-3’) and 805R (16S_805R, 5’-GACTACCAGGGTATCTAATC-3’) primers to amplify the V3-V4 region of the 16S rDNA gene [25]. The sequencing data (fastq) for this study are accessible in the ENA (European Nucleotide Archive, https://www.ebi.ac.uk/ena) database under the PRJEB55863 (ERP140807) bioproject.

### Bioinformatics analysis of 16S amplicons

For this study, we performed all diversity analyses using an amplicon sequence variant (ASV) detection approach (DADA2 pipeline), but we also compared the diversity with an OTU clustering method (based on FROGs, [26]).

For ASV analysis, raw amplicon barcoding data were demultiplexed and processed using the Bioconductor Workflow for Microbiome Data analysis [27]. This workflow is based on DADA2 [12] that infers amplicon sequence variants (ASV) from raw sequence reads. Forward and reverse reads were trimmed at 20 bp, respectively, to remove primers and adapters, and then quality-truncated at 280 and 205 bp, respectively. The dada2 denoise-paired function with default parameters was used to correct sequencing errors and infer exact amplicon sequence variants (ASVs). Then forward and reverse corrected reads were merged with a minimum 20 bp overlap, and the *removeBimeraDenovo* function from DADA2 was used to remove chimeric sequences. Eighty-two percent of reads passed chimeric filtering. The numbers of reads filtered, merged and non-chimeric are indicated in S2 Table. A mean of 58.6% of reads passed all filters (denoising, merging, non-chimeric), with a minimum of 15,347 and a maximum of 31,134 reads in filtered libraries, yielding a total of 2,712 ASV.

ASV were then assigned at the taxonomic level using the DADA2 *AssignTaxonomy* function, with the Silva 16S reference database (silva_nr_v132_train_set) [28]. We subsequently filtered out plasts (especially mitochondria from root samples) to keep only ASVs assigned to the Bacteria or Archaea kingdoms. A last filtering was done to remove ASV with <10 read occurrence across all libraries. A dataset of 1,647 ASV was used for subsequent diversity analyses. A Neighbour-joining phylogenetic tree of the 1,647 ASV was constructed using MEGA11 [29] by first aligning ASV sequences with MUSCLE [30] and then building a Neighbour joining-tree based on a distance matrix corrected with the Kimura 2P method. Metadata and ASV tables and the phylogenetic tree were uploaded to the NAMCO server for downstream microbiota diversity analyses (https://exbio.wzw.tum.de/namco/, [31]. NAMCO is a microbiome explorer server based on a set of R packages, including Phyloseq for diversity analyses [32] and PICRUSt2 for functional predictions [18]. Alpha-diversity analyses (observed richness, Shannon and Simpson diversity, statistical test with pairwise *post-hoc* Dunn test) were performed with Phyloseq and tidyverse, ggpubr, rstatix, multcompView R packages and plotted with ggplot2. Beta-diversity (NMDS, PERMANOVA) was performed with Phyloseq and Vegan. PICRUSt2 functional predictions were performed to infer metabolic capacities from our 16S amplicon ASV. Functions were predicted in three classes: enzyme classification (EC), KEGG orthology (KO) and molecular pathways (PW). Data were normalised with relative abundance, and a Kruskal-Wallis test was performed across conditions (medium used for CDA and CIA) with the ALDEx2 package [33]. Circular phylogenetic tree annotations and mapping were obtained with iTOL [34]. Additional R scripts for the DADA2 pipeline, Phyloseq, and the production of figures are freely available on GitHub (https://github.com/lmoulin34/Article_Moussa_culturingbias).

For the OTU clustering approach, the FROGs pipeline ([26]; http://frogs.toulouse.inra.fr/) was used in the Galaxy environment. After demultiplexing and pre-processing, reads were clustered into OTU using the swarming method with default parameters (aggregation distance of 3), then chimeric sequences were removed and OTU were affiliated with taxonomic levels using the same Assign taxonomy tool as described above.

## Results

### Quality filtering and diversity indices of 16S amplicon libraries (CIA versus CDA)

**Fig 2:**
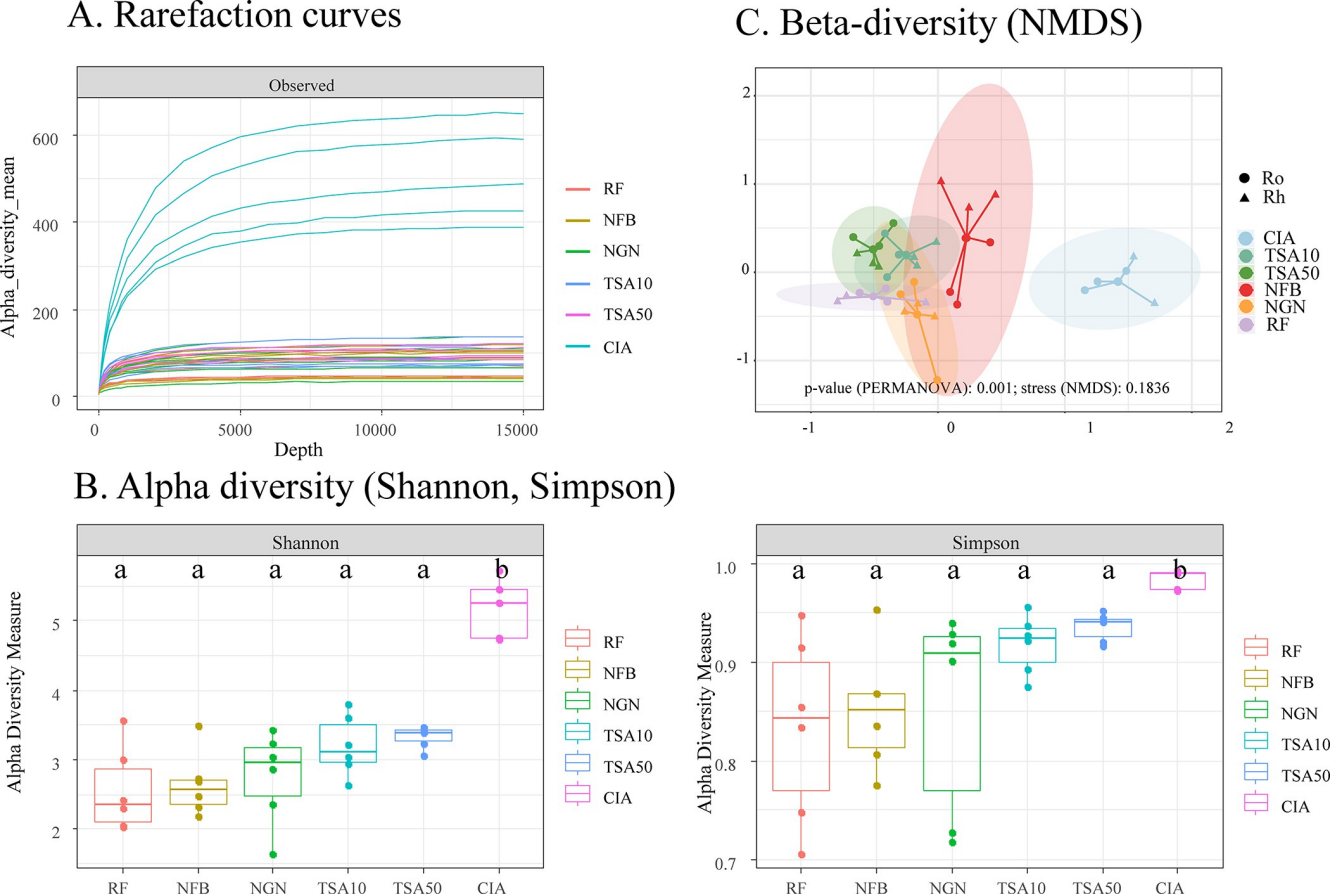
Rarefaction curves (A), alpha (B) and beta diversity (C) in 16S amplicon libraries. Alpha diversity (A) rarefaction curves were calculated on unfiltered data, while alpha diversity indices (B) were calculated using the Shannon and Simpson index. Letters above boxplots indicate statistical significance using a pairwise Wilcoxon test (adjusted with the Bonferroni method). The p-value of a PERMANOVA test on beta diversity is indicated at the bottom of (C). Coloured circles on the NDMS are ellipses of confidence (at 95%) for all media and CIA conditions. Abbreviations: CIA: culture-independent approach; media used in the CDA: RF: rice flour, NFB: nitrogen-free medium, NGN: Norris glucose N-free medium, TSA10 and TSA50: tryptone soya agar at 10 and 50%.

We first assessed the quantity and quality of reads produced for each amplicon library originating from direct rice root or rhizosphere genomic DNA extraction (CIA) or from DNA extracted from cultures (CDA) of the same samples grown on bacterial culture media. A range of 24,000 to 44,000 reads (mean 36,120) was obtained for all 16S amplicon libraries (S2 Table). Rarefaction curves (Fig 2A) showed sampled diversity saturation for each library, with a clear difference between the CIA reads (much higher in alpha diversity) compared to CDA.

After DADA2 pipeline processing, we obtained 2,712 amplicon sequence variants (ASV) that were assigned at the taxonomic level using the Silva database. One library (S36) was removed from the analysis (from CIA) as it showed only 3 ASV. For the remaining libraries, ASV were filtered with regards to their abundance (cumulated reads ≥ 10 among all libraries) and mitochondria, chloroplast and eukaryote reads were removed (remaining ASV = 1647).

We first compared the diversity obtained from root (Ro) and rhizosphere (Rh) samples. There was no statistical difference in ASV alpha diversity (Shannon index) or beta diversity (PERMANOVA) between Ro and Rh samples (S1 Fig). These results could be explained by the fact that we did not surface disinfect or remove the rhizoplane from roots, so the rhizosphere (soil adhering to roots) and the root (rhizoplane + endosphere) from the same samples did not show significant differences. As the focus of this study was to compare the diversity obtained from a non-culturable versus a culturable approach on different media, we pooled Rh and Ro data from the same plant samples for all subsequent analyses.

The bacterial sequences obtained by the CIA method exhibit significantly higher alpha diversity than those obtained from the five CDA media (TSA10, TSA50, NGN, NFb, RF) (Shannon or Simpson index, Kruskal-Wallis test, p = 0,002; Fig 2B). The TSA, RF and nitrogen-free media alpha diversities were not statistically different (Fig 2B). The ASV richness sampled from each medium represented about 15% of the diversity of all ASV detected in both CIA and CDA (TSA10: 16%, TSA50: 14.9%, NFb: 17%, NGN: 17%), except for RF (11%) which captured less diversity, while the CIA approach represented 67%. NMDS on the beta diversity analyses showed no overlap between ASV obtained from the different media (CDA) and the CIA (Fig 2C, PERMANOVA, R^2^ (Linear fit) = 0,88, p = 0.001). A substantial overlap was observed for TSA10 and TSA50, which was expected since it was the same medium but used at two different concentrations.

### Culturable sampled diversity: Comparison between ASV and OTU

We also analysed our amplicon barcoding reads using an OTU-clustering approach (FROGs pipeline, using the swarming method to merge reads into OTU). This approach produced 1,023 OTU after quality filtering (same as for the ASV analysis). We then assessed if the diversity obtained by OTU gave the same percentage diversity recovery compared to ASV. In Table 1, we present the number of ASV and OTU obtained from the culture-dependent approach (CDA) and from the culture-independent approach (CIA), as well as the number of classes, orders and families represented in each. The ASV analysis produced more richness (38% more) than the OTU analysis. This higher diversity was observed at different taxonomic levels: class (ASV:50; OTU:38), order (ASV: 124; OTU:67), and families (ASV:219; OTU:119). Given this result, we conducted all subsequent analyses with ASV-analysed data as it was better at capturing the diversity of our 16S amplicon libraries. In both analyses, the diversity shared between CDA and CIA was relatively low (7% for ASV, 22% for OTU). From the culturable approach, we thus recovered many bacterial taxa that were undetected in the amplicon sequencing performed on gDNA extracted from roots or the rhizosphere, yet only a small proportion of the root bacteria were able to grow on our culture media.

**Table 1:** Comparison of diversity in culture-dependent (CDA) and culture-independent (CIA) approaches, using ASV or OTU analysis.

Taxonomic level	Total number	CIA	CDA	Shared	Specific CIA	Specific CDA
ASV	1,647	1,078	658	128	986	533
OTU	1,023	650	601	228	422	373
% of total ASV	100	65.4	39.9	7.8	59.8	32.4
% of total OTU	100	63.5	58.7	22.3	41.2	36.5
Class (ASV/OTU)	50/38	49/37	12/7	11/11	38/26	1/1
%	100	98/97.3	24.5/18.4	22/29	76/68.4	2/2.6
Order (ASV/OTU)	124/67	121/64	34/28	31/24	90/40	3/3
%	100	97.5/95.5	27.4/41.8	25/35.8	72.6/59.7	2.4/4.5
Family(ASV/OTU)	219/119	203/107	63/54	47/42	156/65	16/12
%	100	92.7/89.9	28.8/45.4	21.5/35.3	71.2/54.6	7.3/10.1

The analysis was performed on ASV and OTU filtered ≥ 10 reads (cumulated among all libraries).

### Comparison of bacterial taxonomic diversity between culture-independent (CIA) and culture-dependent (CDA) approaches

**Fig 3:**
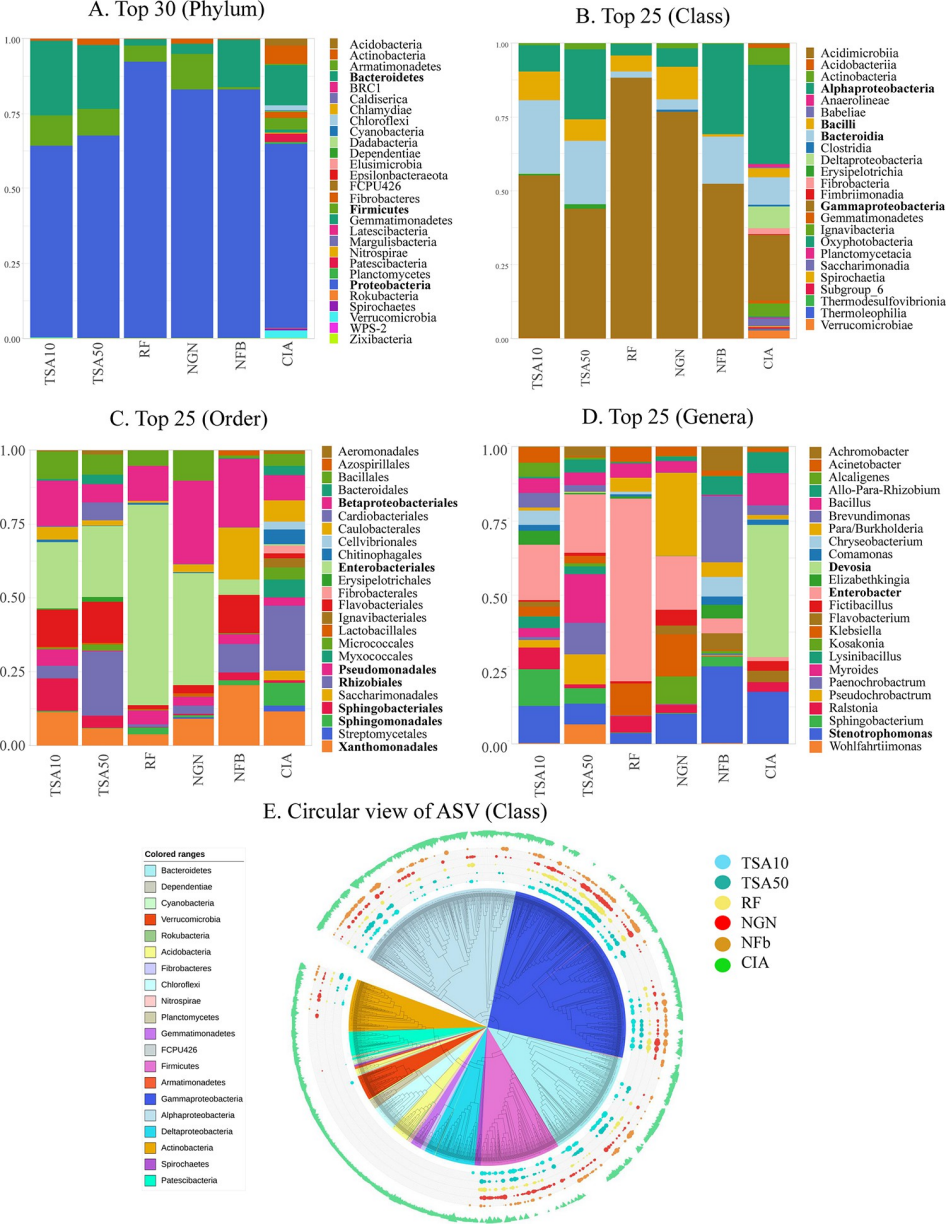
Taxonomic binning of ASV. Taxonomic binning at phylum (A, top 30), class (B, top 25), order (C, top 25) and genus (D, top 25) levels (with most abundant taxa in bold), and a circular phylogenetic tree of ASV with the class-rank distribution among CIA and CDA (E).

Taxonomic binning was performed at different taxonomic levels for the top 30 phyla and the top 25 classes, orders and genera (Fig 3A–3D). The phylum distribution showed a dominance of Proteobacteria, Bacteroidetes and Firmicutes in all libraries, with a clearly higher diversity of phyla in the CIA samples. We identified 22 bacterial phyla in the rice root sample microbiota, with only 5 present in the CDA (Proteobacteria, Firmicutes, Bacteroidetes, Actinobacteria, Verrucomicrobia). The proteobacteria phylum was the most abundant in all samples, with a greater proportion noted on the rice flour culture medium. At the class level, the difference in diversity was even more visible with Gammaproteobacteria, Alphaproteobacteria and Bacteroidia dominating in the CDA, while high class diversity was present in the CIA (Fig 3B). At the order level, the CIA showed (as expected) high diversity, while the CDA data were dominated by Enterobacteriales, Betaproteobacteriales, Rhizobiales and Flavobacteriales. Finally, in the top 25 genera, differences among CDA libraries clearly appeared, with the exception of the *Enterobacter* genus which was enriched in all (although to a lesser extent for NFb) (Fig 3D). In the CIA, *Devosia* was the most represented genus. To better visualize the sampled diversity distribution, we built a phylogenetic tree of ASV (diversity labelled at the class level) and mapped their distribution and abundance in the different conditions (coloured outer circles) (Fig 3E). This representation clearly highlights which taxa diversity is sampled and over-represented with the media used in the CDA (e.g. Gammaproteobacteria in blue or Firmicutes in pink), and which whole parts of bacterial diversity were missed compared to the CIA (e.g. Patescibacteria, Armatimonadetes, Deltaproteobacteria, Planctomycetes, Chloroflexi).

### Statistical differential analyses between CIA and CDA at class and genus levels

We performed a Kruskal-Wallis test (ɑ = 0.05, with the Bonferroni multiple test correction method) to identify classes of bacteria with significant differences among CDA and CIA conditions. The statistical test identified 45 classes of bacteria above the significance cut-off level (p<0,05), 37 of which were present only in the CIA (S3 Table), including in the top 10 most frequent class taxa: Ignavibacteria, Saccharimonadia, Fibrobacteria and Acidobacteria. Four classes were present in both the CIA and CDA: Alphaproteobacteria, Gammaproteobacteria, Bacteroidia and Actinobacteria, with Alphaproteobacteria and Gammaproteobacteria being the most represented in the CIA and CDA, respectively (also visible in Fig 3B and 3C).

**Fig 4:**
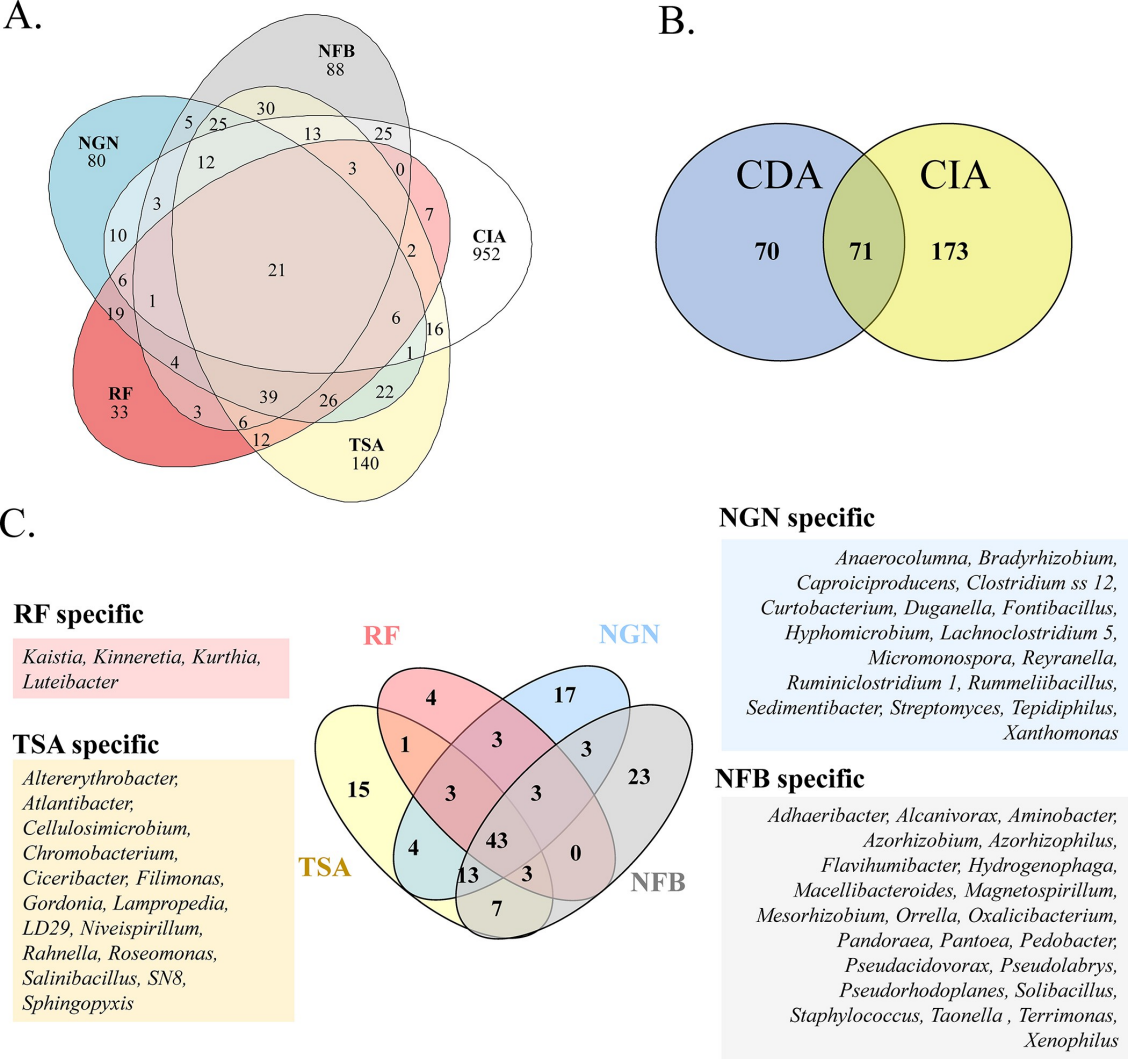
Venn diagrams of the diversity between CDA and CIA. Venn diagrams were produced at the ASV level (A), genus level (B), and between culture media used for the CDA (C). Specific genera obtained on a given culture medium are listed in (C).

Then we performed differential analyses on the mean relative abundance of bacterial genera in each condition, using a Kruskal-Wallis test (ɑ = 0.05). Table 2 shows the 50 most abundant genera in the CIA and their mean relative abundance in each media dataset (whole dataset is available in S4 Table). Among the 20 most frequent bacterial genera in the CIA, eleven were detected in the CDA. These were *Devosia* (8.25% of all genera), obtained on TSA10, TSA50 or NFB media; followed by *Pseudoxanthomonas* (3.62%), which was found in all media conditions except RF, then *Stenotrophomonas* (3.36%), *Bacillus* (2.29%), *Pseudomonas* (1.42%) and *Allo*/*Neo*/*Para*/*Rhizobium* (1.3%) found in all media; and finally *Sphingopyxis* (2.1%) detected in TSA50; Streptomyces (1.48%) in NGN and *Pseudolabrys* (1.47%) in NFb. We built Venn diagrams on shared and specific diversity at ASV (Fig 4A) and genus levels (Fig 4B and 4C). Among the 244 genera from the CIA, 173 (71%) were absent from the culturable approach, while 71 were shared (29%) and 70 others were CDA-specific (Fig 4B). We also compared the genus diversity sampled in each CDA medium, and we listed specific genera obtained for each media on the Venn diagram in Fig 4C.

**Table 2:** List of the top 50 most abundant bacterial genera in the culture-independent approach (CIA), and their occurrence in media of the culturable-dependent approach (CDA).

Genus	CIA	TSA10	TSA50	NFB	NGN	RF	p_value
g__Devosia	8.25	0.03	0.21	0.06	0.00	0.00	2.27E-04
g__Pseudoxanthomonas	3.62	0.14	0.08	0.22	0.01	0.00	1.13E-03
g__Asticcacaulis	3.49	0.00	0.00	0.00	0.00	0.00	2.68E-06
g__70(f__BIrii41)	3.38	0.00	0.00	0.00	0.00	0.00	8.15E-05
g__Stenotrophomonas	3.36	11.13	5.54	20.19	8.69	3.34	3.18E-02
g__Bacillus	2.29	4.43	3.56	0.32	3.26	4.29	2.51E-02
g__Sphingopyxis	2.10	0.00	0.00	0.00	0.00	0.00	1.74E-05
g__Cellvibrio	2.00	0.00	0.00	0.00	0.00	0.00	2.68E-06
g__129(o__OPB56)	1.91	0.00	0.00	0.00	0.00	0.00	2.68E-06
g__126(f__PHOS-HE36)	1.78	0.00	0.00	0.00	0.00	0.00	2.68E-06
g__124(o__Saccharimonadales)	1.55	0.00	0.00	0.00	0.00	0.00	2.68E-06
g__Streptomyces	1.48	0.00	0.00	0.00	0.50	0.00	1.12E-04
g__Pseudolabrys	1.47	0.00	0.00	1.02	0.00	0.00	6.12E-05
g__75(f__Fibrobacteraceae)	1.45	0.00	0.00	0.00	0.00	0.00	2.68E-06
g__Pseudomonas	1.42	0.80	0.01	2.05	0.26	0.22	3.78E-02
g__Allor/Neo/Para/Rhizobium	1.30	0.48	3.60	4.81	1.15	0.37	1.98E-02
g__Rhizomicrobium	1.25	0.00	0.00	0.00	0.00	0.00	8.15E-05
g__Geobacter	1.24	0.00	0.00	0.00	0.00	0.00	8.15E-05
g__Sphingobium	1.23	0.35	0.00	0.03	0.01	0.25	5.23E-03
g__Altererythrobacter	1.22	0.00	0.00	0.00	0.00	0.00	1.74E-05
g__Mesorhizobium	1.04	0.00	0.00	0.01	0.00	0.00	3.73E-05
g__WCHB1-32	1.00	0.00	0.00	0.00	0.00	0.00	8.15E-05
g__Flavisolibacter	0.96	0.00	0.00	0.00	0.00	0.00	8.15E-05
g__125(f__Saccharimonadaceae)	0.92	0.00	0.00	0.00	0.00	0.00	2.68E-06
g__57(f__Microbacteriaceae)	0.91	0.00	0.00	0.00	0.01	0.00	5.10E-04
g__Magnetospirillum	0.87	0.00	0.00	0.00	0.00	0.00	9.72E-03
g__Sphingomonas	0.79	0.00	0.04	1.54	0.22	0.15	1.46E-02
g__38(f__Rhizobiaceae)	0.77	0.00	0.06	0.24	0.21	0.01	6.04E-03
g__Actinotalea	0.73	0.00	0.00	0.00	0.00	0.00	8.15E-05
g__Opitutus	0.73	0.00	0.00	0.00	0.00	0.00	2.68E-06
g__Flavobacterium	0.72	1.38	0.07	4.26	2.62	0.00	4.00E-02
g__122(p__Patescibacteria)	0.67	0.00	0.00	0.00	0.00	0.00	2.68E-06
g__134(f__Chitinophagaceae)	0.65	0.00	0.00	0.00	0.00	0.00	2.68E-06
g__26(f__Reyranellaceae)	0.62	0.00	0.00	0.00	0.00	0.00	2.68E-06
g__Bradyrhizobium	0.61	0.00	0.00	0.00	0.49	0.00	8.98E-05
g__Thermomonas	0.61	0.00	0.00	0.00	0.00	0.00	2.68E-06
g__Paludibaculum	0.60	0.00	0.00	0.00	0.00	0.00	2.68E-06
g__Brevundimonas	0.60	4.38	1.88	17.20	0.18	0.00	1.88E-02
g__Acidovorax	0.60	0.00	0.00	0.00	0.00	0.00	2.68E-06
g__Caulobacter	0.60	0.03	0.07	0.20	2.56	0.72	4.01E-02
g__Parvibaculum	0.59	0.00	0.00	0.00	0.00	0.00	2.68E-06
g__18(o__R7C24)	0.57	0.00	0.00	0.00	0.00	0.00	2.68E-06
g__Hydrogenophaga	0.52	0.00	0.00	0.01	0.00	0.00	5.10E-04
g__Anaeromyxobacter	0.51	0.00	0.00	0.00	0.00	0.00	2.68E-06
g__Aeromonas	0.50	0.30	1.31	0.07	0.00	0.05	9.62E-03
g__143(f__Prolixibacteraceae)	0.50	0.00	0.00	0.00	0.00	0.00	3.03E-02
g__Phycicoccus	0.46	0.00	0.00	0.00	0.00	0.00	3.03E-02
g__Fluviicola	0.46	0.00	0.00	0.00	0.00	0.00	2.68E-06
g__Lacunisphaera	0.44	0.00	0.00	0.00	0.00	0.00	2.68E-06
g__Hylemonella	0.44	0.00	0.00	0.00	0.00	0.00	8.15E-05

Colors indicate frequencies of occurrence in the 16S amplicon data (highest in red, lowest in green, for each medium and CIA condition). Kruskal-Wallis test, ɑ = 0.05.

To document genera that were the most frequent in the culturable approach for each medium, a table of the 20 most statistically frequent genera (Kruskal-Wallis test, ɑ = 0.05) obtained for each medium of the CDA is given in Table 3. In this top 20 most frequent genera, several appeared in all media: *Enterobacter*, *Stenotrophomonas*, *Bacillus*, *Sphingobacterium*, *Klebsiella*, *Brevundimonas* and *Rhizobium*, all of which are known to be fast-growers on rich media and reported to contain plant-inhabiting species. On nitrogen-free media, species known as nitrogen-fixing Plant Growth Promoting Rhizobacteria (PGPR) were sampled: *Azospirillum*, *Para*/*Burkholderia*, *Bradyrhizobium*, *Sphingomonas*, etc.

**Table 3:** Distribution and mean relative abundance of the top 20 genera detected in the CDA.

%	TSA10	%	TSA50	%	RF	%	NFB	%	NGN
16.7	Enterobacter	16.0	Enterobacter	55.7	Enterobacter	20.2	Stenotrophomonas	24.4	Burkholderia s.l.
11.1	Sphingobacterium	13.3	Myroides	9.8	Klebsiella	17.2	Brevundimonas	15.7	Enterobacter
11.1	Stenotrophomonas	8.8	Paenochrobactrum	4.4	Burkholderia s.l.	5.3	Chryseobacterium	12.4	Klebsiella
4.4	Bacillus	8.1	Pseudochrobactrum	4.2	Bacillus	4.8	Rhizobium s.l	8.6	Stenotrophomonas
4.3	Brevundimonas	5.5	Stenotrophomonas	3.3	Stenotrophomonas	4.2	Flavobacterium	3.2	Bacillus
4.3	Chryseobacterium	5.4	Wohlfahrtiimonas	1.9	Novosphingobium	3.7	Enterobacter	2.6	Flavobacterium
4.0	Alcaligenes	4.2	Sphingobacterium	1.1	Proteus	3.7	Burkholderia s.l.	2.5	Caulobacter
3.5	Lysinibacillus	3.6	Rhizobium s.l	0.7	Caulobacter	2.4	Sphingobacterium	1.1	Rhizobium s.l
2.7	Klebsiella	3.5	Bacillus	0.7	Chryseobacterium	2.0	Pseudomonas	0.73	Mycobacterium
2.6	Myroides	3.1	Dysgonomonas	0.5	9(Enterobacteriaceae)	1.7	Azospirillum	0.50	Streptomyces
2.2	Pseudochrobactrum	2.5	Morganella	0.3	Rhizobium s.l	1.5	Sphingomonas	0.49	Bradyrhizobium
1.3	Flavobacterium	2.2	Lysinibacillus	0.2	Sphingobium	1.0	Pseudolabrys	0.34	Novosphingobium
1.0	Morganella	1.9	Klebsiella	0.2	Pseudomonas	0.9	Xanthobacter	0.26	Pseudomonas
0.9	Burkholderia s.l.	1.8	Brevundimonas	0.1	Sphingomonas	0.9	Massilia	0.25	Sphingobacterium
0.8	Paenochrobactrum	1.8	Leucobacter	0.1	Sphingobacterium	0.6	Ochrobactrum	0.22	Sphingomonas
0.8	Pseudomonas	1.6	Erysipelothrix	0.05	Aeromonas	0.3	Paenochrobactrum	0.22	Hyphomicrobium
0.6	Bordetella	1.3	Aeromonas	0.04	Xanthobacter	0.3	Bacillus	0.21	38(f__Rhizobiaceae)
0.4	Rhizobium s.l	0.8	Bordetella	0.04	Ancylobacter	0.3	Klebsiella	0.18	Brevundimonas
0.4	Erysipelothrix	0.6	Ignatzschineria	0.04	Paenalcaligenes	0.2	38(f__Rhizobiaceae)	0.15	Xanthobacter
0.4	Dysgonomonas	0.6	Vagococcus	0.03	Myroides	0.2	Alcaligenes	0.11	9(Enterobacteriaceae)

Genera present in at least 4 media are coloured. Only significant genera are shown (Kruskal-Wallis test, ɑ = 0.01).

### Prediction of enriched functions in CIA compared to culture-based approach

**Fig 5:**
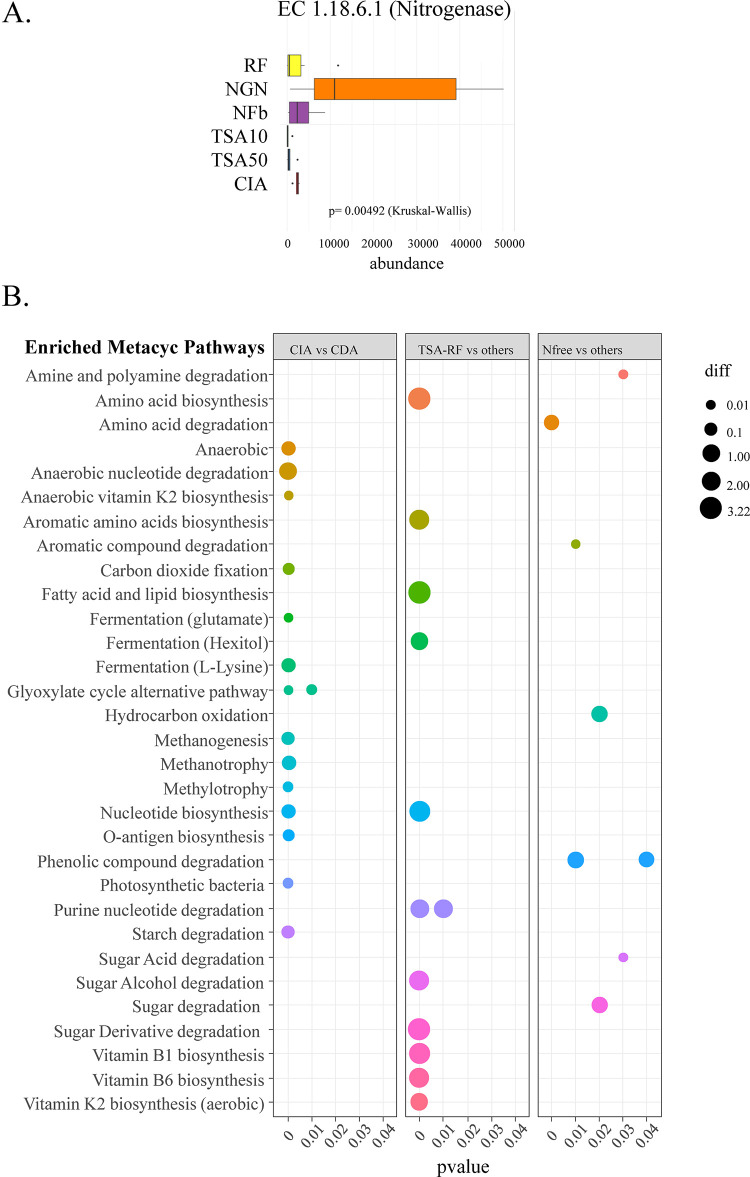
Predicted enriched pathways in amplicon libraries. Nitrogenase enrichment prediction in 16S amplicon libraries (A) and dot-plot of predicted enriched Metacyc pathways (B) in CIA vs CDA cross comparisons, non-selective media (TSA, RF) versus others, and nitrogen-free media versus others. Enzyme and pathway enrichment were predicted with PICRUSt2 and a differential analysis was performed with a Kruskal-Wallis test. “diff” is the abbreviation for differential analysis (see Materials & Methods).

We performed a functional prediction analysis using PICRUSt2 to infer metabolic capacities from our 16S amplicon ASV. In order to assess the predictive ability of the PICRUSt2 algorithm on our dataset, we focused on the specific enzyme nitrogenase (EC. 1.18.6.1) prediction in CDA libraries that included medium with (TSA, RF) or without nitrogen (NGN, NFb) (Fig 5A). As expected, we observed nitrogenase enrichment (p = 0.00492) in nitrogen-free NFb and NGN media, with NGN medium exhibiting much higher enrichment than NFb. The non-selective medium (TSA) and the plant-based medium (RF) did not enrich bacterial taxa with the nitrogenase function (Fig 5A).

We also aimed to predict which functional pathways were specific to CIA compared to CDA in order to help design conditions to capture the yet unculturable diversity. We thus analysed the metabolic pathways (based on PW/Metacyc categories) predicted as being enriched in the CIA compared to CDA conditions, and represented the results in a dot-plot (Fig 5B). Among the detected Metacyc pathways enriched in CIA, several functions linked to specific ecological niche abilities were detected: anaerobic/fermentation metabolism, carbon dioxide fixation, bacterial photosynthesis, methanotrophy and methylotrophy. As our CDA culture conditions were aerobic and in the dark, this enrichment was logical and gave clues on the culture conditions that could ultimately capture more bacterial diversity. Enriched pathways in the TSA and RF media libraries (compared to others) could be linked to heterotrophy on rich media in aerobic conditions (sugar degradation, amino acid/lipid/nucleotide biosynthesis, vitamin biosynthesis). For the nitrogen-free media (compared to the others), several pathways were detected as phenolic compound/polyamine/amino acid degradation and sugar degradation. Nitrogen fixation does not appear as itself in Metacyc pathways, it is embedded in “nitrogen metabolism” together with “nitrification” and “denitrification” capacities among others, so that no pattern from nitrogen fixation ability is possible, apart from the analysis on E.C. for the nitrogenase enzyme (Fig 5A).

## Discussion

In this study we used Illumina sequencing on 16S-amplicon barcodes (variable region V3-V4) to quantify the bacterial diversity bias that occur when culturing rice root-associated bacteria on a range of culture media compared to the real diversity. Our goal was to precisely document the bacterial taxa diversity that could be recovered from a set of culture media compared to the real diversity, the predicted and enriched/depleted functions that could be inferred from this diversity, while seeking how to design new culture conditions to capture it. We have used the term “real diversity” in reference to that captured by Illumina amplicon sequencing, although this approach can also be biased, since it is based on the amplification of a marker gene from a DNA matrix that could originate from dead bacteria. As rice is a non-perennial plant and we expected root and rhizosphere soil to be under high metabolic turnover, we hypothesized that DNA from dead bacteria in the culture-independent approach would not represent high diversity in our analysis. Yet this is a possibility and could represent bias from the unculturable approach when comparing culture and uncultured-based diversity analyses.

Several studies have already compared culturable and real rice microbiota diversity [35–37], but they often relied on comparisons between regular 16S Sanger sequencing on isolated bacteria compared to NGS sequences. Here we used the same sequencing methodology at high depth and were able to compare diversity levels without sequencing/analytical bias. We also used two different analytical methods to infer operational taxonomic units, i.e. ASV (based on exact sequence variant detection, [12] or OTU (based on clustering by swarming, [11]). As ASV analysis detected more diversity than OTU at different levels (even class and order levels, Table 1), we preferred to use ASV for all subsequent diversity and functional predictions. As exact sequence variants highly rely on algorithms for sequencing error detection and correction, we could not exclude that some of the obtained diversity was due to algorithm imperfections. However, given that the obtained higher diversity also concerned higher taxonomic levels, this artificial diversity issue is unlikely as it would involve a high number of mutations.

In this study, the diversity obtained from the CDA culture media (TSA10, TSA50, RF, NGN, NFb) was lower compared to the CIA. If we combine all diversity of the CDA, it represents 11.7% (ASV level), 29% (genus level), 22.4% (class level), 25.6% (order level) and 23.1% (family level) of the diversity of the CIA. As there are few comparable studies in the literature, it is hard to determine if our recovery rate was low or high since this is the first study to our knowledge to have assessed culturable recovery by amplicon barcoding and NGS sequencing. The review of Sarhan et al. [16] detailed recent advances in culturomics methodologies, and established a recovery rate of about 10% for conventional chemically-synthetic culture media, which is in the range that we obtained at the ASV level (although we obtained 23 to 29% at higher taxonomic levels). Samson et al. [36] claimed to have recovered up to 70% of bacterial genera (on 16S V5-V7 amplicon, at >97% similarity) from *Oryza sativa indica* and *japonica* rice microbiota, but they applied a 0.1% frequency cut-off. Applying the same cut-off on our dataset would indicate the detection of 121 genera in the CIA, with 36 (29%) of them present in the CDA.

From all media used in the CDA, we could recover a total of 142 bacterial genera, with each medium capturing 15 to 23 specific genera (Fig 4C). The only exception was the plant-based rice flour medium, which in our study captured low bacterial diversity compared to the other media, probably due to its low composition complexity. Plant-based media have been suggested to be a good alternative to popular bacterial chemical media for increasing the cultivability of plant-associated microbes [16], but the use of homogenised roots, leaves or exudates has been recommended to complement minimal or more complex media.

The recovery of specific ASV from the CDA that were not detected in the CIA was an unexpected finding in our study. This diversity represented 532 ASV, 1 class, 3 orders, 16 families and 70 genera (Table 1, Fig 4A and 4B). This number of ASV in CDA may seem high (the total number in both CIA and CDA was 1,647), but it only represented a quarter of the total ASV diversity at the class, order and family levels (Table 1). We cannot exclude that a technical PCR bias could have increased the diversity from the CDA, since DNA polymerase errors may arise. Moreover, if there is low diversity in the DNA matrix, a diverse range of sequence variants could be produced, but these errors would only affect diversity at species or genus levels in the amplicon sequencing, not at higher taxonomic levels as in our results. One explanation for not detecting, in the CIA, the ASV diversity found in CDA could concern the sequencing depth, yet the rarefaction curves did reach a plateau but at much higher alpha diversity for the CIA compared to the CDA (Fig 2A). The mean sequencing depth obtained was 36,120. If differences between bacterial ASV frequencies exceed 10e^4^, then several genera may be undetected in the CIA approach, whereas they may be selected by specific culturable media. We set the read number filter at 10 (cumulated in all libraries), but we also looked at lower filtering (>2) and unfiltered ASV data (S5 Table). In the unfiltered data, we counted 102 specific bacterial genera for CDA, 243 for CIA, with 90 in common, while these numbers were 70, 173 and 71 in the filtered results (10 read filter, Table 1 and Fig 4B), i.e. similar proportions. Processing unfiltered data thus produced similar proportions of specific ASV for the CDA compared to CIA. Regardless of the filtering method, we detected one specific class in CDA (undetected in CIA), i.e. Erysipelotrichia, represented by one genus, i.e. *Erysipelothrix*, and 4 ASV recovered from TSA medium (at 10 and 50% concentration). A BLAST study of these ASV sequences revealed 100% sequence identity with the 16S rDNA of *Erysipelothrix inopinata*—a species whose type strain was isolated from sterile-filtered vegetable broth [38]. As our medium was sterilized by autoclaving, it is unlikely that these 4 ASV were contaminants. It should also be noted that we are not the first to have found that isolates from culturable approaches were undetected in culture-independent approaches [14, 37]. It would be better to assess rice microbiota diversity by substantially increasing the sequencing depth in order to get a better image of the overall diversity. The frequency differences between ASV exceeding 10e^3^ (at the genus level, S4 Table) that we found in our study means that they would not be detected in the CIA, while they would be in the culture-based approach. This was a crucial finding since several studies have underlined the role of rare species (also called satellite taxa) in plant-microbe interactions and more broadly in key ecosystem functions [13, 39]. Increasing the representativeness of taxonomic diversity in databases should also be the focus of further scientific research since many ASV cannot be affiliated to taxonomic ranks due to missing descriptions of these taxa in taxonomic databases.

We also tried to predict functions and metabolic pathways that would be enriched when using different types of media, and we also conducted statistical tests to highlight functions that were missing from our culturable approach. Metagenome-guided isolation and cultivation of microbes has been developed in recent years, but these approaches are based on metagenomic sequences and the reconstruction of genomes and metabolic pathways [19]. The massive sequencing effort focused on a highly diverse range of bacteria from different environments has led to the development of genome database and prediction tools that may be used with simple amplicon taxonomic markers [18]. We applied a prediction tool to our dataset to investigate the ecology and functional capacities of our detected bacteria. We found that many taxa with anaerobic metabolisms such as methanogenesis (methane production), methanotrophy (methane degradation) and methylotrophy (one-carbon reduction), or with photosynthetic capacities, were missing from CDA (Fig 5) compared to the CIA. It is well known that rice microbiota differ from microbiota of other crops since rice is often grown in flooded conditions, thereby creating an oxic-anoxic interface between the rhizosphere/root system and the bulk soil [4, 5]. Our functional prediction approach thus underlined the presence of these probably strictly anaerobic bacteria adapted to anoxic conditions in the CIA, and their absence from the CDA. These predictions provided clues on the specific conditions and compositions of media required to capture these yet unculturable functional groups of bacteria. They could also serve to develop culturomics, a growing scientific field for microbiologists interested in synthetic microbiota and for biotechnological applications of plant-associated microorganisms.

## Supporting information

S1 Fig Boxplot of the Shannon alpha diversity index (1A) and NMDS of beta-diversity (1B) of root and rhizosphere 16S amplicon libraries. (DOCX)

S1 Table Dilutions and incubation times used for DNA extraction in the culturable approach. (DOCX)

S2 Table 16S amplicon statistics in the DADA2 pipeline. (DOCX)

S3 Table Abundance of bacterial classes detected in rice microbiota (Wilcoxon test), and their occurrence in the culturable approach. Numbers are colored according to class frequencies in each medium and CIA amplicon data.
(XLSX)

S4 Table Abundance of the 264 bacterial genera detected in rice microbiota (Kruskal-Wallis test), and their occurrence in the culturable approach. (XLSX)

S5 Table ASV count table with taxonomic ranks. The Excel file contains: sheet 1, unfiltered data, sheets 2 &3, filtered at 2 and 10 cumulated reads in all libraries, respectively.
(XLSX)

## References

[1] Bulgarelli D, Schlaeppi K, Spaepen S, van Themaat EVL, Schulze-Lefert P. Structure and Functions of the Bacterial Microbiota of Plants. Annu Rev Plant Biol. 2013;64: 807–838. doi: 10.1146/annurev-arplant-050312-120106 23373698

[2] Simonin M, Dasilva C, Terzi V, Ngonkeu ELM, Diouf D, Kane A, et al. Influence of plant genotype and soil on the wheat rhizosphere microbiome: evidences for a core microbiome across eight African and European soils. FEMS Microbiol Ecol. 2020;96: fiaa067. doi: 10.1093/femsec/fiaa067 32275297

[3] Edwards J, Johnson C, Santos-Medellín C, Lurie E, Podishetty NK, Bhatnagar S, et al. Structure, variation, and assembly of the root-associated microbiomes of rice. Proc Natl Acad Sci U S A. 2015;112: E911–E920. doi: 10.1073/pnas.1414592112 25605935 PMC4345613

[4] Ding L-J, Cui H-L, Nie S-A, Long X-E, Duan G-L, Zhu Y-G. Microbiomes inhabiting rice roots and rhizosphere. FEMS Microbiol Ecol. 2019;95: fiz040. doi: 10.1093/femsec/fiz040 30916760

[5] Kim H, Lee YH. The rice microbiome: A model platform for crop holobiome. Phytobiomes J. 2020;4: 5–18. doi: 10.1094/PBIOMES-07-19-0035-RVW/ASSET/IMAGES/LARGE/PBIOMES-07-19-0035-RVWF2.JPEG

[6] Barro M, Wonni I, Simonin M, Kassankogno AI, Klonowska A, Moulin L, et al. The impact of the rice production system (irrigated vs lowland) on root-associated microbiome from farmer’s fields in western Burkina Faso. FEMS Microbiol Ecol. 2022;98: fiac085. doi: 10.1093/femsec/fiac085 35867879

[7] Barret M, Briand M, Bonneau S, Préveaux A, Valière S, Bouchez O, et al. Emergence shapes the structure of the seed microbiota. Appl Environ Microbiol. 2015;81: 1257–1266. doi: 10.1128/AEM.03722-14 25501471 PMC4309697

[8] Ogier JC, Pagès S, Galan M, Barret M, Gaudriault S. RpoB, a promising marker for analyzing the diversity of bacterial communities by amplicon sequencing. BMC Microbiol. 2019;19. doi: 10.1186/s12866-019-1546-z 31357928 PMC6664775

[9] Getzke F, Hacquard S. High-Throughput Profiling of Root-Associated Microbial Communities. Methods Mol Biol Clifton NJ. 2022;2494: 325–337. doi: 10.1007/978-1-0716-2297-1_23 35467218

[10] Mahé F, Rognes T, Quince C, de Vargas C, Dunthorn M. Swarm: robust and fast clustering method for amplicon-based studies. PeerJ. 2014;2: e593. doi: 10.7717/peerj.593 25276506 PMC4178461

[11] Mahé F, Czech L, Stamatakis A, Quince C, Vargas C de, Dunthorn M, et al. Swarm v3: towards tera-scale amplicon clustering. Bioinformatics. 2021;38: 267–269. doi: 10.1093/bioinformatics/btab493 34244702 PMC8696092

[12] Callahan BJ, McMurdie PJ, Rosen MJ, Han AW, Johnson AJA, Holmes SP. DADA2: High-resolution sample inference from Illumina amplicon data. Nat Methods. 2016;13: 581–583. doi: 10.1038/nmeth.3869 27214047 PMC4927377

[13] Compant S, Samad A, Faist H, Sessitsch A. A review on the plant microbiome: Ecology, functions, and emerging trends in microbial application. J Adv Res. 2019;19: 29–37. doi: 10.1016/j.jare.2019.03.004 31341667 PMC6630030

[14] Bai Y, Müller DB, Srinivas G, Garrido-Oter R, Potthoff E, Rott M, et al. Functional overlap of the Arabidopsis leaf and root microbiota. Nature. 2015;528: 364–369. doi: 10.1038/nature16192 26633631

[15] de Souza RSC, Armanhi JSL, Arruda P. From Microbiome to Traits: Designing Synthetic Microbial Communities for Improved Crop Resiliency. Front Plant Sci. 2020;11. Available: https://www.frontiersin.org/articles/10.3389/fpls.2020.01179 32983187 10.3389/fpls.2020.01179 PMC7484511

[16] Sarhan MS, Hamza MA, Youssef HH, Patz S, Becker M, ElSawey H, et al. Culturomics of the plant prokaryotic microbiome and the dawn of plant-based culture media–A review. J Adv Res. 2019;19: 15–27. doi: 10.1016/j.jare.2019.04.002 31341666 PMC6630032

[17] Zhang J, Liu Y-X, Guo X, Qin Y, Garrido-Oter R, Schulze-Lefert P, et al. High-throughput cultivation and identification of bacteria from the plant root microbiota. Nat Protoc. 2021;16: 988–1012. doi: 10.1038/s41596-020-00444-7 33442053

[18] Douglas GM, Maffei VJ, Zaneveld JR, Yurgel SN, Brown JR, Taylor CM, et al. PICRUSt2 for prediction of metagenome functions. Nat Biotechnol. 2020;38: 685–688. doi: 10.1038/s41587-020-0548-6 32483366 PMC7365738

[19] Liu S, Moon CD, Zheng N, Huws S, Zhao S, Wang J. Opportunities and challenges of using metagenomic data to bring uncultured microbes into cultivation. Microbiome. 2022;10: 76. doi: 10.1186/s40168-022-01272-5 35546409 PMC9097414

[20] Liu X, Wang M, Nie Y, Wu X-L. Isolation Chip Increases Culturable Bacterial Diversity and Reduces Cultivation Bias. Curr Microbiol. 2021;78: 2025–2032. doi: 10.1007/s00284-021-02474-0 33821359

[21] Youseif SH, Abd El-Megeed FH, Humm EA, Maymon M, Mohamed AH, Saleh SA, et al. Comparative Analysis of the Cultured and Total Bacterial Community in the Wheat Rhizosphere Microbiome Using Culture-Dependent and Culture-Independent Approaches. Microbiol Spectr. 2021;9: e00678–21. doi: 10.1128/Spectrum.00678-21 34668733 PMC8528112

[22] Baldani JI, Reis VM, Videira SS, Boddey LH, Baldani VLD. The art of isolating nitrogen-fixing bacteria from non-leguminous plants using N-free semi-solid media: a practical guide for microbiologists. Plant Soil. 2014;384: 413–431. doi: 10.1007/S11104-014-2186-6

[23] Ranganayaki S, Mohan C. Effect of sodium molybdate on microbial fixation of nitrogen. Z Allg Mikrobiol. 1981;21: 607–610. 7331378

[24] Odjo T, Diagne D, Adreit H, Milazzo J, Raveloson H, Andriantsimialona D, et al. Structure of African Populations of Pyricularia oryzae from Rice. Phytopathology®. 2021;111: 1428–1437. doi: 10.1094/PHYTO-05-20-0186-R 33386066

[25] Sinclair L, Osman OA, Bertilsson S, Eiler A. Microbial Community Composition and Diversity via 16 S rRNA Gene Amplicons: Evaluating the Illumina Platform. PLoS ONE. 2015;10: e0116955. doi: 10.1371/journal.pone.0116955 25647581 PMC4315398

[26] Escudié F, Auer L, Bernard M, Mariadassou M, Cauquil L, Vidal K, et al. FROGS: Find, Rapidly, OTUs with Galaxy Solution. Bioinformatics. 2018;34: 1287–1294. doi: 10.1093/bioinformatics/btx791 29228191

[27] Callahan BJ, Sankaran K, Fukuyama JA, McMurdie PJ, Holmes SP. Bioconductor Workflow for Microbiome Data Analysis: from raw reads to community analyses. F1000Research. 2016;5: 1492. doi: 10.12688/f1000research.8986.2 27508062 PMC4955027

[28] Quast C, Pruesse E, Yilmaz P, Gerken J, Schweer T, Yarza P, et al. The SILVA ribosomal RNA gene database project: improved data processing and web-based tools. Nucleic Acids Res. 2013;41: D590–D596. doi: 10.1093/nar/gks1219 23193283 PMC3531112

[29] Tamura K, Stecher G, Kumar S. MEGA11: Molecular Evolutionary Genetics Analysis Version 11. Mol Biol Evol. 2021;38: 3022–3027. doi: 10.1093/molbev/msab120 33892491 PMC8233496

[30] Edgar R. MUSCLE: multiple sequence alignment with high accuracy and high throughput. Nucleic Acids Res. 2004;32: 1792–7. doi: 10.1093/nar/gkh340 15034147 PMC390337

[31] Dietrich A, Matchado MS, Zwiebel M, Ölke B, Lauber M, Lagkouvardos I, et al. Namco: a microbiome explorer. Microb Genomics. 2022;8: mgen000852. doi: 10.1099/mgen.0.000852 35917163 PMC9484756

[32] McMurdie PJ, Holmes S. Phyloseq: An R Package for Reproducible Interactive Analysis and Graphics of Microbiome Census Data. PLoS ONE. 2013;8. doi: 10.1371/journal.pone.0061217 23630581 PMC3632530

[33] Fernandes AD, Reid JN, Macklaim JM, McMurrough TA, Edgell DR, Gloor GB. Unifying the analysis of high-throughput sequencing datasets: characterizing RNA-seq, 16S rRNA gene sequencing and selective growth experiments by compositional data analysis. Microbiome. 2014;2: 15. doi: 10.1186/2049-2618-2-15 24910773 PMC4030730

[34] Letunic I, Bork P. Interactive tree of life (iTOL) v5: An online tool for phylogenetic tree display and annotation. Nucleic Acids Res. 2021;49: W293–W296. doi: 10.1093/nar/gkab301 33885785 PMC8265157

[35] Bertani I, Abbruscato P, Piffanelli P, Subramoni S, Venturi V. Rice bacterial endophytes: isolation of a collection, identification of beneficial strains and microbiome analysis. Environ Microbiol Rep. 2016;8: 388–398. doi: 10.1111/1758-2229.12403 27038229

[36] Zhang J, Liu Y-X, Zhang N, Hu B, Jin T, Xu H, et al. NRT1.1B is associated with root microbiota composition and nitrogen use in field-grown rice. Nat Biotechnol. 2019;37: 676–684. doi: 10.1038/s41587-019-0104-4 31036930

[37] Samson M, Bez C, Georges H, Joseph B, Venturi V. Characterization of bacterial strains from bacterial culture collection of rice sheath in Burundi highlights an Alcaligenes species strain with antibacterial activity against Pseudomonas fuscovaginae rice pathogen. Afr J Microbiol Res. 2021;15: 497–511. doi: 10.5897/AJMR2021.9513

[38] Verbarg S, Rheims H, Emus S, Frühling A, Kroppenstedt RM, Stackebrandt E, et al. Erysipelothrix inopinata sp. nov., isolated in the course of sterile filtration of vegetable peptone broth, and description of Erysipelotrichaceae fam. nov. Int J Syst Evol Microbiol. 2004;54: 221–225. doi: 10.1099/ijs.0.02898-0 14742484

[39] Hol WHG, de Boer W, de Hollander M, Kuramae EE, Meisner A, van der Putten WH. Context dependency and saturating effects of loss of rare soil microbes on plant productivity. Front Plant Sci. 2015;6. Available: https://www.frontiersin.org/articles/10.3389/fpls.2015.00485 26175749 10.3389/fpls.2015.00485 PMC4485053

