# Supplementary material for: Quantification of diversity sampling bias resulting from rice root bacterial isolation on popular and nitrogen-free culture media using 16S amplicon barcoding
Source: PLoS One. 2023 Apr 6;18(4):e0279049. doi: 10.1371/journal.pone.0279049 (PMC10079111; doi:10.1371/journal.pone.0279049)
Supplement: S1 Fig — (DOCX) [file pone.0279049.s001.docx]

**S1 Fig. Boxplot of Shannon alpha diversity index (1A) and NMDS of beta-diversity (1B) of root and rhizosphere 16S amplicon libraries**. .A : Boxplot of Shannon diversity index between Ro and Rh samples  in CDA (culture-dependant) and CIA (culture-independent) conditions. Letters indicate statistical groups from pairwise posthoc test (Dunn test with Bonferroni correction; α=0.05). B. Non-metric multidimensional scaling (NMDS) of ASV beta-diversity. P-value indicated (not significative) is the result of a PERMANOVA test between Ro and Rh samples.

A. Boxplot of Shannon alpha diversity index

a

a

b

b

2

3

4

5

6

CDA-Ro

CDA-Rh

CIA-Ro

CIA-Rh

Shannon

B. NMDS of beta diversity of ASV samples.
