# Supplementary material for: Quantification of diversity sampling bias resulting from rice root bacterial isolation on popular and nitrogen-free culture media using 16S amplicon barcoding
Source: PLoS One. 2023 Apr 6;18(4):e0279049. doi: 10.1371/journal.pone.0279049 (PMC10079111; doi:10.1371/journal.pone.0279049)
Supplement: S1 Table — (DOCX) [file pone.0279049.s002.docx]

Supplementary Table S1. Dilutions and incubation time used for DNA extractions in the culturable approach

| Sample compartment | Culture medium | Dilution used for DNA extraction | | | | Bacterial culture incubation time |
| --- | --- | --- | --- | --- | --- | --- |
|  |  | 10^-1^ | 10^-2^ | 10^-3^ | 10^-4^ | (days) |
| Ro | TSA 10% |  | √ |  |  | 3 |
| Ro | TSA 50% |  |  |  | √ | 2 |
| Ro | NGN |  | √ |  |  | 3 |
| Ro | RF |  | √ |  |  | 2 |
| Ro | NFb | √ |  |  |  | 7 |
| RH | TSA 10% |  |  | √ |  | 3 |
| RH | TSA 50% |  |  | √ |  | 2 |
| RH | NGN |  | √ |  |  | 3 |
| RH | FR |  |  | √ |  | 2 |
| RH | NFb | √ |  |  |  | 7 |
