# Supplementary material for: Quantification of diversity sampling bias resulting from rice root bacterial isolation on popular and nitrogen-free culture media using 16S amplicon barcoding
Source: PLoS One. 2023 Apr 6;18(4):e0279049. doi: 10.1371/journal.pone.0279049 (PMC10079111; doi:10.1371/journal.pone.0279049)
Supplement: S2 Table — (DOCX) [file pone.0279049.s003.docx]

Supplementary Table S2. 16S amplicons statistics in DADA2 pipeline

| Sample Name | Compartment | Medium | Raw reads | filtered | denoisedF | denoisedR | merged | nonchim |
| --- | --- | --- | --- | --- | --- | --- | --- | --- |
| S1 | Ro | TSA10 | 42798 | 33358 | 32801 | 33153 | 31134 | 22927 |
| S2 | Ro | TSA10 | 36665 | 28103 | 27735 | 27964 | 26336 | 19463 |
| S3 | Ro | TSA10 | 38461 | 30004 | 29285 | 29735 | 27181 | 20015 |
| S4 | Ro | TSA50 | 37929 | 30183 | 29247 | 29951 | 26817 | 19583 |
| S5 | Ro | TSA50 | 39059 | 31312 | 30542 | 30964 | 27627 | 20110 |
| S6 | Ro | TSA50 | 37732 | 29622 | 29081 | 29408 | 26632 | 19145 |
| S7 | Ro | NFB | 32260 | 25434 | 25328 | 25360 | 25169 | 25139 |
| S8 | Ro | NFB | 43111 | 33878 | 33513 | 33676 | 32126 | 24512 |
| S9 | Ro | NFB | 38247 | 29628 | 29298 | 29485 | 28190 | 19271 |
| S10 | Ro | NGN | 43078 | 33643 | 33512 | 33569 | 33197 | 26366 |
| S11 | Ro | NGN | 41019 | 31515 | 31103 | 31319 | 30028 | 25113 |
| S12 | Ro | NGN | 38112 | 29292 | 28981 | 29178 | 28367 | 27049 |
| S13 | Ro | RF | 39908 | 31234 | 30761 | 31088 | 29168 | 20985 |
| S14 | Ro | RF | 31039 | 19330 | 19163 | 19298 | 18860 | 16907 |
| S15 | Ro | RF | 35752 | 27883 | 27678 | 27827 | 27299 | 24826 |
| S16 | Rh | TSA10 | 43295 | 33491 | 32245 | 33141 | 28461 | 22341 |
| S17 | Rh | TSA10 | 36280 | 28214 | 27305 | 27908 | 24125 | 18282 |
| S18 | Rh | TSA10 | 33800 | 25696 | 25158 | 25565 | 23412 | 17578 |
| S19 | Rh | TSA50 | 38248 | 30439 | 29743 | 30191 | 27258 | 20278 |
| S20 | Rh | TSA50 | 37026 | 29099 | 28550 | 28901 | 26417 | 22059 |
| S21 | Rh | TSA50 | 33407 | 25378 | 24614 | 25117 | 22354 | 16982 |
| S22 | Rh | NFB | 36656 | 27838 | 27303 | 27633 | 25397 | 22123 |
| S23 | Rh | NFB | 38690 | 30027 | 29479 | 29759 | 27511 | 21333 |
| S24 | Rh | NFB | 44841 | 34970 | 34192 | 34515 | 30226 | 21640 |
| S25 | Rh | NGN | 43293 | 33670 | 33083 | 33471 | 31657 | 25433 |
| S26 | Rh | NGN | 39126 | 30142 | 29686 | 29978 | 28277 | 23296 |
| S27 | Rh | NGN | 41176 | 31346 | 30860 | 31155 | 29569 | 21686 |
| S28 | Rh | RF | 36346 | 28902 | 28386 | 28724 | 26823 | 21582 |
| S29 | Rh | RF | 38069 | 29323 | 29193 | 29293 | 28981 | 27492 |
| S30 | Rh | RF | 32559 | 25507 | 25358 | 25469 | 25071 | 23225 |
| S31 | Rh | CIA | 32709 | 25353 | 23787 | 24431 | 21520 | 20531 |
| S32 | Rh | CIA | 32607 | 25094 | 23473 | 24198 | 21117 | 20190 |
| S33 | Rh | CIA | 28462 | 22216 | 20965 | 21543 | 19161 | 18581 |
| S34 | Ro | CIA | 29042 | 21761 | 19159 | 20113 | 15911 | 15347 |
| S35 | Ro | CIA | 30556 | 22811 | 20304 | 21349 | 17316 | 16814 |
